# Imagery Network Fine Registration by Reference Point Cloud Data Based on the Tie Points and Planes

**DOI:** 10.3390/s21010317

**Published:** 2021-01-05

**Authors:** Mehrdad Eslami, Mohammad Saadatseresht

**Affiliations:** School of Surveying and Geospatial Engineering, College of Engineering, University of Tehran, Tehran 1439957131, Iran; msaadat@ut.ac.ir

**Keywords:** fine registration, photogrammetric imagery, laser scanner point cloud, mobile mapping systems, calibration

## Abstract

Cameras and laser scanners are complementary tools for a 2D/3D information generation. Systematic and random errors cause the misalignment of the multi-sensor imagery and point cloud data. In this paper, a novel feature-based approach is proposed for imagery and point cloud fine registration. The tie points and its two neighbor pixels are matched in the overlap images, which are intersected in the object space to create the differential tie plane. A preprocessing is applied to the corresponding tie points and non-robust ones are removed. Initial coarse Exterior Orientation Parameters (EOPs), Interior Orientation Parameters (IOPs), and Additional Parameters (APs) are used to transform tie plane points to the object space. Then, the nearest points of the point cloud data to the transformed tie plane points are estimated. These estimated points are used to calculate Directional Vectors (DV) of the differential planes. As a constraint equation along with the collinearity equation, each object space tie point is forced to be located on the point cloud differential plane. Two different indoor and outdoor experimental data are used to assess the proposed approach. Achieved results show about 2.5 pixels errors on checkpoints. Such results demonstrated the robustness and practicality of the proposed approach.

## 1. Introduction

Developments in recent decades on laser scanners and digital cameras production have affected the productivity of 2D/3D spatial data generation systems. Meanwhile, the recent development of fast and cheaper computers has influenced the aforementioned data processing. Photogrammetric, computer vision, and remote sensing images are broadly used to generate 2D planimetric maps and land cover classification [1,2], change detection [3], and 3D objects reconstruction [4,5]. Accurate alignment between image scenes, precise self-calibration, noise-less dense point cloud generation, and imaging network design are top challenges of the image-based information generation strategies.

Also, laser scanner point cloud (aerial, Unmanned Aerial Vehicle (UAV), terrestrial, handheld, and mobile) data have largely been used in land cover classification [6], urban building detection and reconstruction [7], and 3D object modelling [8]. There are several challenges with point cloud data in colorizing the points, accurate edges sampling, range accuracy, and the density of the points [9].

As widely accepted criteria, point cloud, and imagery datasets are proved to be complimentary. Many studies have fused these datasets and reported practical results [10,11]. Such fusion needs two wide-ranging steps: Registration (spatial, spectral, and temporal) and integration/combination (pixel, object, or decision). Spatial fine registration/co-registration is a critical step for object detection, 3D reconstruction, object modelling, and other applications [12]. A large number of approaches have been used for fine registration of the point cloud and imagery data, which are categorized as below.

Statistical/area-based approaches: Such approaches refine Exterior Orientation Parameters (EOPs) by minimizing a cost function [13]. These methods renovate 2D-3D to a 2D-2D registration problem by converting the point cloud data to the range-images [14]. In this regard, different cost functions such as correlation function maximization [15], statistical dependence by Mutual Information (MI) [16,17], and adaptive MI-based methods [14,18] are used for fine registration of the various aerial, UAV or close-range imagery, and point cloud data [19]. However, [20] shows that above-mentioned area-based methods are not useful in natural areas. Furthermore, the authors of the [21] have been proved that feature-based approaches are more robust than the statistical/area-based ones. There are broad challenges with these methods to fine registration of the multi modal sensors data while these data have different statistical properties.

Multi-view-based approaches: These approaches alter the point cloud and imagery fine registration problem into a 3D-3D problem. Commonly, Structure from Motion (SfM) and Multi-View Stereo (MVS) methods are implemented for the imagery data and, as a result, an image-based dense point cloud is formed. Then, Iterative Closest Point (ICP) methods are used to register both 3D-3D datasets [12,22]. Further, [23] are used control points and surfaces to register single-modal range images, based on a calculation of a transformation matrix and minimizing control points distance. The methods based on ICP algorithm needs that two datasets to have overlap with each other and local minima is the other problem of the such methods.

Feature-based approaches: Points, lines, and planes are the basic features used in a variety of 2D-3D registration applications. Affine Scale- Invariant Feature Transform (ASIFT) has some features to gain fine registration of the close-range imagery with TLS point cloud data [24]. Center of the buildings [25,26] and also buildings corner [14] are used to fine the registration. The researchers of [27,28] employed collinearity equation and refined the transformation parameters of the images and laser scanner point cloud data. Furthermore, the researchers of [29] employed a point based method, which uses accurate and precise GNSS/INS and Ground Control Point (GCP) to calibrate trajectory data for simultaneously registration of the LiDAR and imagery data. Lines (straight lines and edges) and planes (area, surface, plane, and region) are the other type of features adapted for fine registration. Different methods have been used in various line type features as building roof edges [30], surface intersection lines [31], straight lines [32], and linear map features of the Geospatial Information System (GIS) layers [33] to fine registration of the imagery and point cloud data. Correspondingly, the authors of [34] used the segmented point cloud and imagery data semantic plane features based on Particle Swarm Optimization (PSO) approach to fine mobile mapping laser scanner and cameras imagery data registration. Buildings shadow regions and sensors metadata are the other information adopting for satellite images registration to the LiDAR point cloud [35].

Occlusions of the point, line, and plane features as well as finding correct corresponding features in the imagery and point cloud data are the most important complexity and challenges involved in using the feature-based approaches. To the best of our knowledge, there is not a complete method to overcome all above-mentioned problems and challenges for multi-view based area/statistical-based and feature-based approaches.

In this paper, a novel hybrid point and plane feature-based approach is proposed for coarse-to-fine registration of a variety of the indoor and outdoor photogrammetric imagery and laser scanner point cloud datasets.

The aim of the coarse-to-fine registration approach is to estimate Interior Orientation Parameters (IOPs), Additional Parameters (APs), and EOPs for all of the sensors. Our method assumes that all these parameters (i.e., IOPs, Aps, and EOPs) are known for the laser scanner. Also, it is assumed that The EOPs, IOPs, and especially the APs should be explicitly given.

The approach proposed in this study does not need finding any corresponding point features. This approach employs imagery every tie point and its neighbor pixels to build a photogrammetric object spaceplane. Then, the nearest point cloud plane to the photogrammetric one is estimated. It must be noted that we do not claim that our method could work in the multipart green regions such as forest areas.

The basic concept is to force object space photogrammetric tie point to be located as possible as on its corresponding point cloud plane. Such an idea will occur by adding the constraint equation along with the collinearity equation and applying Least Square Adjustment (LSA) to estimate accurate unknown parameters. The main differences with the methods of the [27,28] are that how the local plane is determined and gross errors are eliminated. Another good feature of the proposed method is the use of surface normal deviations between the photogrammetric and laser scanner data.

The remainder of this paper is organized as follows. In Section 2, methods and materials are discussed. Section 3 contains experimental data and implementation on two different indoor and outdoor data. Section 4 contains analyzing and discussions. Finally, Section 5 presents the conclusions.

## 2. Methods and Materials

**Figure 1:**
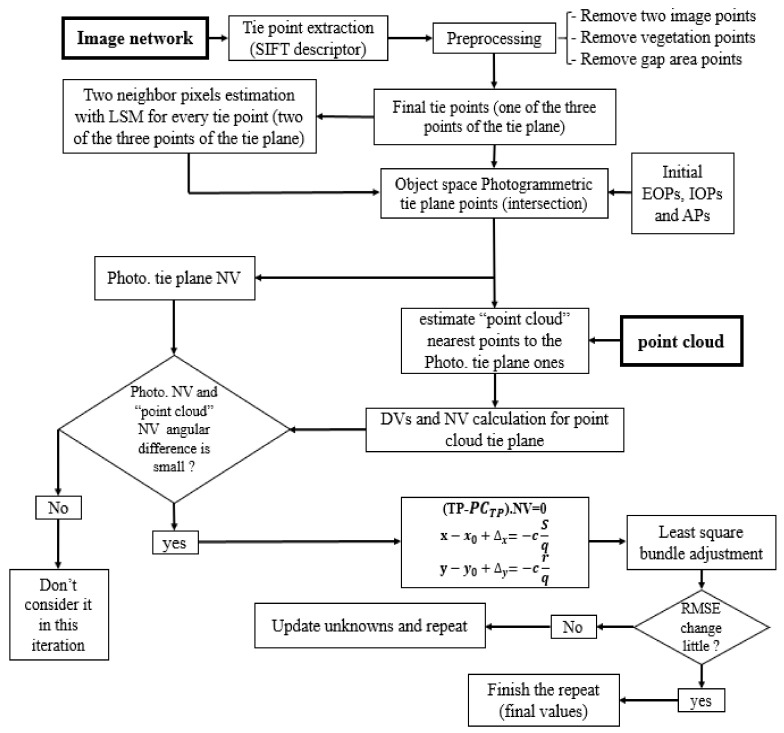
Flowchart of the proposed approach.

To overcome the misalignment and non-calibration between photogrammetric image network and laser scanner point cloud data, a comprehensive novel approach is proposed. A general flowchart of our method is shown in Figure 1. The proposed approach uses a tie points as initial control feature. Next, plane features are created, and the constraint equation based on tie points and planes is established beside the collinearity equation for coarse-to-fine registration as follows.

### 2.1. Tie Point Extraction and Tie Plane Estimation

Scale-Invariant Feature Transform (SIFT) [36] is applied to extract corresponding tie points in all imagery data. To gain robust and precise tie points, three preprocessing steps are applied to the initial tie points. First, tie points that are matched just in two images are removed. Second, a Support Vector Machine (SVM) [37] method is utilized to classify vegetation type classes. Afterward, the tie points on such class are removed. Finally, the tie points are used to build tie planes. Here, it is a reality that vegetation is an object type that is dynamic, non-stable, and non-flat objects.

**Figure 2:**
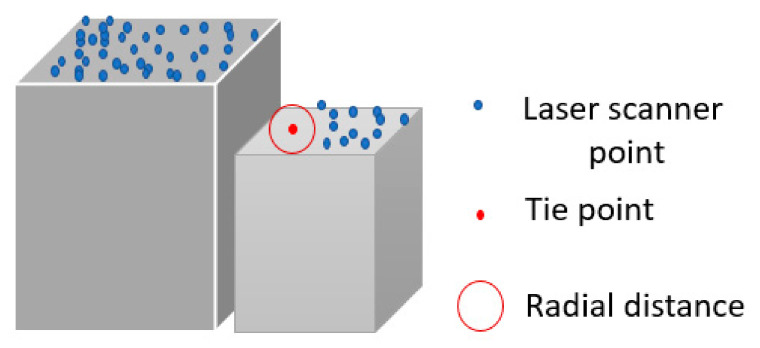
Tie points in occluded area of the laser scanner point cloud.

Third, tie points are transformed by the space intersection algorithm to the object space and a radius is defined to find the neighboring laser scanner points. For this purpose, we selected 20 GSDs range distance based on the coarse registration accuracy and the trial and error. The tie points located in the occluded areas of the point cloud data are eliminated (Figure 2).

Here, we assume that these parameters are known for all laser scan stations and that inaccurate EOPs, IOPs, and APs of images are estimated via coarse registration. This inaccurate EOPs, IOPs, and APs values are coming from the approximate sensor position or using inaccurate and a limited number of control points.

**Figure 3:**
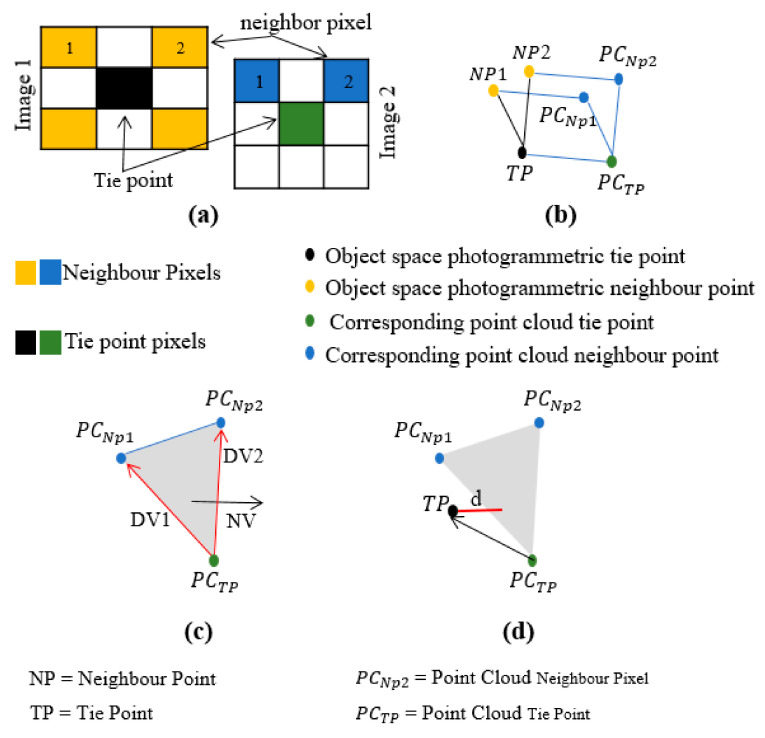
Tie points and least square matching to find neighbor pixels (**a**), object space photogrammetric and point cloud tie and neighbor pixels (**b**), tie plane, Directional Vectors (DVs) and Normal Vector (NV) estimation (**c**), and closest photogrammetric tie point to the tie plane (**d**).

After preprocessing, for each remained tie point, two neighbor pixels were selected and matched in all overlapped images (Figure 3a based on least square matching [38] method. These two neighbor pixels and their tie point are transformed by collinearity space intersection equation to the object space. These three points make a photogrammetric plane in object space. For each point of the photogrammetric plane, the nearest point of the point cloud ones is estimated (Figure 3b).

Therefore, we have three points of the point cloud data that are used to build a tie plane. Also, based on three estimated tie plane points, a Normal Vector (NV) from Directional Vectors (DV) can be built as follows (Figure 3c).

(1) $$\mathrm{DV}1=\left[\begin{array}{c} X_{{\mathrm{PC}}_{\mathrm{NP}1}}\\ Y_{{\mathrm{PC}}_{\mathrm{NP}1}}\\ Z_{{\mathrm{PC}}_{\mathrm{NP}1}} \end{array}\right]-\left[\begin{array}{c} X_{{\mathrm{PC}}_{\mathrm{TP}}}\\ Y_{{\mathrm{PC}}_{\mathrm{TP}}}\\ Z_{{\mathrm{PC}}_{\mathrm{TP}}} \end{array}\right]$$

(2) $$\mathrm{DV}2=\left[\begin{array}{c} X_{{\mathrm{PC}}_{\mathrm{NP}2}}\\ Y_{{\mathrm{PC}}_{\mathrm{NP}2}}\\ Z_{{\mathrm{PC}}_{\mathrm{NP}2}} \end{array}\right]-\left[\begin{array}{c} X_{{\mathrm{PC}}_{\mathrm{TP}}}\\ Y_{{\mathrm{PC}}_{\mathrm{TP}}}\\ Z_{{\mathrm{PC}}_{\mathrm{TP}}} \end{array}\right]$$

where $X_{{\mathrm{PC}}_{\mathrm{TP}}}$, $Y_{{\mathrm{PC}}_{\mathrm{TP}}}$, and $Z_{{\mathrm{PC}}_{\mathrm{TP}}}$ are the of the point cloud tie point. Also, $X_{{\mathrm{PC}}_{\mathrm{NP}1}}$, $Y_{{\mathrm{PC}}_{\mathrm{NP}1}}$,$Z_{{\mathrm{PC}}_{\mathrm{NP}1}}$, $X_{{\mathrm{PC}}_{\mathrm{NP}2}}$, $Y_{{\mathrm{PC}}_{\mathrm{NP}2}}$, and $Z_{{\mathrm{PC}}_{\mathrm{NP}2}}$ are the coordinates of the point cloud neighbour points. The NV can be estimated as the cross product of the two aforementioned DVs (3).

(3) NV=DV1×DV2

### 2.2. Constraint Equations and Bundle Adjustment

If the photogrammetric IOPs, EOPs, and APs are correctly estimated, the object space photogrammetric tie point will be located on the point cloud tie plane or will be as close as possible to it. The distance d (Figure 3d) should be close to zero. Because of the coarse registration, the distance d is not zero.

(4) $${\mathrm{DV}}_{\mathrm{TP}-{\mathrm{PC}}_{\mathrm{TP}}}=\left[\begin{array}{c} X_{\mathrm{TP}}\\ Y_{\mathrm{TP}}\\ Z_{\mathrm{TP}} \end{array}\right]-\left[\begin{array}{c} X_{{\mathrm{PC}}_{\mathrm{TP}}}\\ Y_{{\mathrm{PC}}_{\mathrm{TP}}}\\ Z_{{\mathrm{PC}}_{\mathrm{TP}}} \end{array}\right]$$

We use this concept to add a new constraint equation along with the collinearity equation for coarse-to-fine registration. The ${\mathrm{DV}}_{\mathrm{TP}-{\mathrm{PC}}_{\mathrm{TP}}}$ (the directional vector) between object space photogrammetric tie point and its corresponding point cloud point is built as (4), where $X_{\mathrm{TP}}$, $Y_{\mathrm{TP}}$, and $Z_{\mathrm{TP}}$ are the coordinates of the object space photogrammetric tie point.

Therefore, to force the photogrammetric tie point to be located on the tie plane, the shift constraint equation used (5). For each tie point, the shift constraint equation will add one equation to the final bundle adjustment equation.

(5) $$\mathrm{CE}={\mathrm{DV}}_{\mathrm{TP}-{\mathrm{PC}}_{\mathrm{TP}}}•\mathrm{NV}$$

As a straightforward photogrammetric main geometry in case of distortion free imaging, each point in the object space (3D point), corresponding image space point (2D point), and the perspective center of the camera are collinear, which is named collinearity condition. The collinearity equation is defined to every tie points as below:

(6) $$\left[\begin{array}{c} x-x_{0}\\ y-y_{0}\\ -c \end{array}\right]+\Delta c=\lambda R\left[\begin{array}{c} X_{\mathrm{TP}}\\ Y_{\mathrm{TP}}\\ Z_{\mathrm{TP}} \end{array}\right]+\left[\begin{array}{c} X_{T}\\ Y_{T}\\ Z_{T} \end{array}\right]$$

where x and y are the image coordinate of the tie point. $x_{0}$, $y_{0}$, and c are the IOPs (principle point coordinates and principle distance) of the camera. R is the rotation matrix between the image principal coordinate system and laser scanner reference coordinate system (7) and λ is the scale between them.

(7) $$R=\left[\begin{array}{ccc} R_{11} & R_{12} & R_{13}\\ R_{21} & R_{22} & R_{23}\\ R_{31} & R_{32} & R_{33} \end{array}\right]$$

$X_{T}$, $Y_{T}$, and $Z_{T}$ are the coordinates of the image principal center with respect to the laser scanner reference coordinate system. Δc is an equation that contains APs of the camera as (8).

(8) $$\left\{\begin{array}{c} \Delta x=\left(x-x_{0}\right)\left(L_{1}r^{2}+L_{2}r^{4}\right)+O_{1}[r^{2}+2\left(x-x_{0}{)}^{2}\right]+2O_{2}\left(x-x_{0}\right)\times \left(y-y_{0}\right)\\ \Delta y=\left(y-y_{0}\right)\left(L_{1}r^{2}+L_{2}r^{4}\right)+O2[r^{2}+2\left(y-y_{0}{)}^{2}\right]+2O_{1}\left(x-x_{0}\right)\times \left(y-y_{0}\right) \end{array}\right.$$

where $L_{1}$, $L_{2}$, $O_{1}$, and $O_{2}$ are the lens radial and tangential distortion parameters.

(9) $$r=\sqrt{{\left(x-x_{0}\right)}^{2}+{\left(y-y_{0}\right)}^{2}}$$

By simplifying Equation (6), final collinearity equation can be formulated as (10).

(10) $$\left\{\begin{array}{c} \left(x-x_{0}\right)+\Delta x=-c\frac{R_{11}\left(X_{\mathrm{TP}}-X_{T}\right)+R_{12}\left(Y_{\mathrm{TP}}-Y_{T}\right)+R_{13}\left(Z_{\mathrm{TP}}-Z_{T}\right)}{R_{31}\left(X_{\mathrm{TP}}-X_{T}\right)+R_{32}\left(Y_{\mathrm{TP}}-Y_{T}\right)+R_{33}\left(Z_{\mathrm{TP}}-Z_{T}\right)}\\ \left(y-y_{0}\right)+\Delta y=-c\frac{R_{21}\left(X_{\mathrm{TP}}-X_{T}\right)+R_{22}\left(Y_{\mathrm{TP}}-Y_{T}\right)+R_{23}\left(Z_{\mathrm{TP}}-Z_{T}\right)}{R_{31}\left(X_{\mathrm{TP}}-X_{T}\right)+R_{32}\left(Y_{\mathrm{TP}}-Y_{T}\right)+R_{33}\left(Z_{\mathrm{TP}}-Z_{T}\right)} \end{array},\right.$$

where Equations (5) and (10) are applied to each tie point for fine registration of the imagery and point cloud data. These equations consist of four groups of the unknowns: EOPs, IOPs, Aps, and tie points object space coordinates.

The common photogrammetric bundle adjustment procedure [39] is applied to obtain the iterative least squares estimation process. The error equations are expanded by employing the first-order Taylor series as follows:

(11) $$\left\{\begin{array}{c} V_{\mathrm{CE}}=E_{1}\Delta P-Z_{1}\\ V_{\mathrm{PCE}}=X_{1}\Delta T+X_{2}\Delta P+X_{3}\Delta I-Z_{2}\\ V_{\mathrm{AP}}=S_{1}\Delta I-Z_{3} \end{array}\right.$$

where $V_{\mathrm{CE}}$, $V_{\mathrm{PCE}}$, and $V_{\mathrm{AP}}$ are the residual vectors of the expanded Equations (5), (8), and (10). $X_{1}$, $X_{2}$, $X_{3}$, $E_{1}$, and $S_{1}$ are the partial derivatives with respect to the unknowns (i.e., EOPs, IOPs, Aps, and object coordinates of the tie points). ΔP, ΔT, and ΔI are the correction values and $Z_{1}$, $Z_{2}$, and $Z_{3}$ are the constant values.

## 3. Experimental Data and Implementation

In this section, our proposed approach is implemented on two different outdoor and indoor datasets. Also, we discussed the study dataset.

### 3.1. Study Dataset

**Figure 4:**
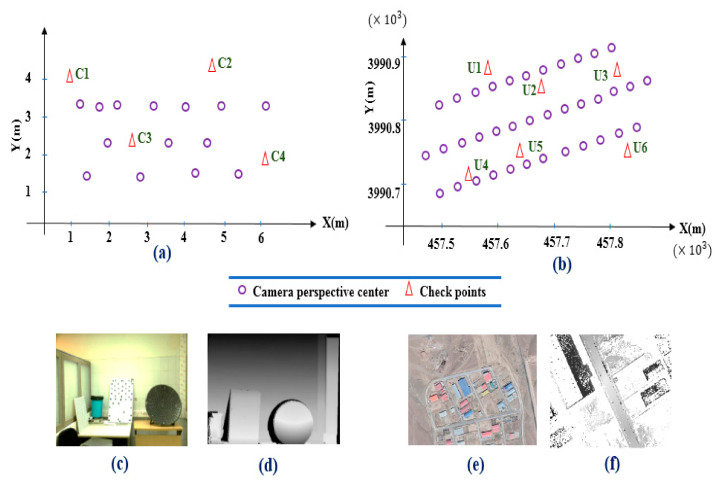
Position of the camera perspective center of the close-range imagery data (**a**) and Unmanned Aerial Vehicle (UAV) data (**b**). Study area of the close-range data (**c**) and UAV data (**e**). Point cloud for close-range data (**d**) and UAV data (**f**).

Our experimental dataset contains two different close-range and UAV photogrammetry data (Figure 4). Close-range imagery data consist of 14 images (Figure 4c) that are captured by Canon 7D camera and point cloud data are generated by the Leica ScanStation2 laser scanner (Figure 4d). The Ground Sampling Distance (GSD) value for close-range imagery data is 0.001 m and density of the point cloud data is 180,000 points per meter.

Also, UAV data contain 37 images captured by DJI phantom 4 pro camera sensor in three flight lines of 60% overlap (lateral overlap) and 80% of overlap between images (forward overlap) in an industrial region in the Abyek city of the Qazvin Province, Iran (Figure 4e). RIEGL VMX-250 laser scanner system is used to generate point cloud data (Figure 4f). Further information about datasets is illustrated in Table 1. The GSD value for UAV imagery data is 0.025 m and the flight altitude of the UAV drone is 80 m over the region. Further, the density of the point cloud data is 40,000 points per meter.

Furthermore, as an assumption our method considers that coarse registration is done. So, we used not well-distributed and a limited number of GCPs to coarsely register both datasets.

### 3.2. Implementation

**Figure 5:**
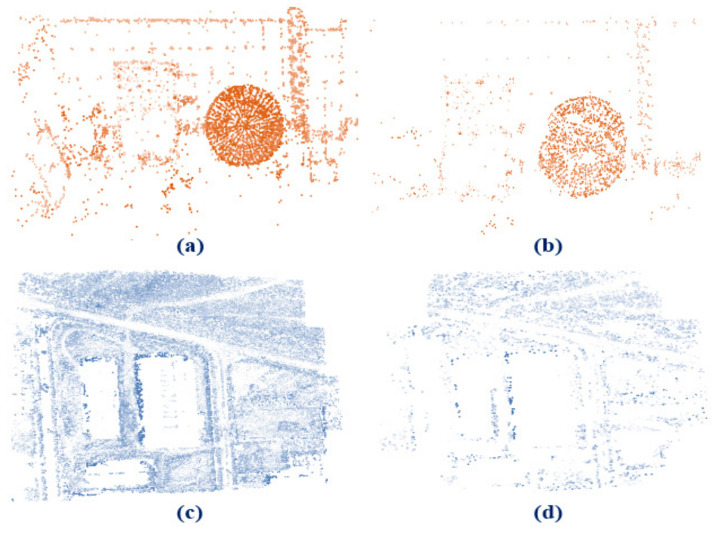
Initially extracted tie points for close-range (**a**) and UAV (**c**) dataset before applying preprocessing step. Remaining tie points after applying preprocessing step for close-range (**b**) and UAV (**d**) datasets.

As mentioned before, SIFT descriptor is used to produce tie points from study dataset. As shown in Figure 5a,c, initial 220,647 and 4084 corresponding tie points for UAV and close-range data are generated correspondingly.

Afterward, tie points matching in the two images are removed. So, the number of tie points is reduced to 94,281 and 1220, respectively. Next, tie points are reduced to 10,002 and 921 points, respectively, after removing point cloud occluded areas and vegetation preprocessing steps for UAV and close-range datasets (Figure 5b,d). Tie points will be used in the next steps to generate tie planes. Vegetation includes highly non-stable, dynamic, and non-flat objects. We used the SVM method to mask out tie points distinguished in the vegetated areas.

Using the remained final corresponding tie points of the two datasets, NVs, DVs, and planes are produced. Then, NVs are used to remove tie points that cannot pass the gross error analysis step. Furthermore, if photogrammetric and the corresponding point cloud planes NVs angularly are very different, then tie points are removed. After that, the proposed method and bundle adjustment procedure are done as follows:

1. Initial EOPs, APs, and IOPs values are specified. The initial non-accurate values of the IOPs, APs, and EOPs can come from the approximate sensor positioning systems or adopting non-accurate and a low number of the control points (in this paper the coarse values of the EOPs for UAV and close-range datasets are from GNSS and GCP values, respectively. Also, the IOPs, APs are from initial bundle adjustment of the imagery network by using the beforementioned EOPs values).
2. Search for closest point cloud points to the object space photogrammetric tie points (if the all distances (d) average is small enough, then stop procedure; otherwise continue).
3. Fit a plane to the found points in Step 2 and estimate the DVs and NVs.
4. Compare the photogrammetric and the corresponding point cloud planes NVs. Remove the gross tie points and its planes.
5. Organize the observation equations (i.e., the error equations).
6. Solve least square bundle adjustment procedure to acquire corrections to the EOPs, IOPs, APs values, corrections to the object coordinates of tie points and update the unknown parameters.
7. Calculate the Root Mean Square Error (RMSE) for all corrections of the unknown values.
8. Check if the RMSE value is less than the threshold (which was selected to be 0.01 by trial and error) go back to step 2; otherwise, repeat from step 6.

Iterative calculations lead to the ultimate EOPs, APs, and IOPs values. Quality and accuracy assessment of the fine registration proposed method between imagery and point cloud data is a crucial step.

## 4. Analyzing and Discussion

The common traditional process to assess our coarse-to-fine registration method is to use well-distributed checkpoints. So, precise and accurate measured checkpoints are employed to analyze the quality of our method. Figure 4a,b illustrate the well-distributed checkpoints (4 and 6 points for close range and UAV data, respectively) position on the study dataset. The checkpoints for the UAV data are located on the buildings and ground and for the close range on the different objects. Also, the checkpoints are identifiable in both image and point cloud data. RMSE, minimum, average, and maximum errors are calculated for checkpoints before and after applying the proposed method. The differences between the check point coordinates are computed in the object space and converted to pixels using the GSD given in Table 1.

Total (i.e., the XYZ) coarse registration minimum, average, and maximum errors on the close-range data were measured to be about 19.32, 29.67, and 39.04 pixels, respectively. Meanwhile, total fine registration minimum, average, and maximum errors were improved to 1.91, 2.15, and 2.51 pixels, respectively. These values on UAV data were measured to be about 14.58, 20.5, and 25.81 pixels, respectively, and then improved to 1.61, 2.26, and 2.57 pixels for minimum, average, and maximum error values, respectively. Furthermore, the RMSE value on close-range and UAV data before applying the fine registration method is about 30.60 and 20.85 pixels, respectively. These values change to 2.25 and 2.28 pixels, respectively, after applying the fine registration method. In Table 2, a comparison between the coarse and fine registration maximum and average accuracies and RMSE values for the close range and UAV data is illustrated.

**Figure 6:**
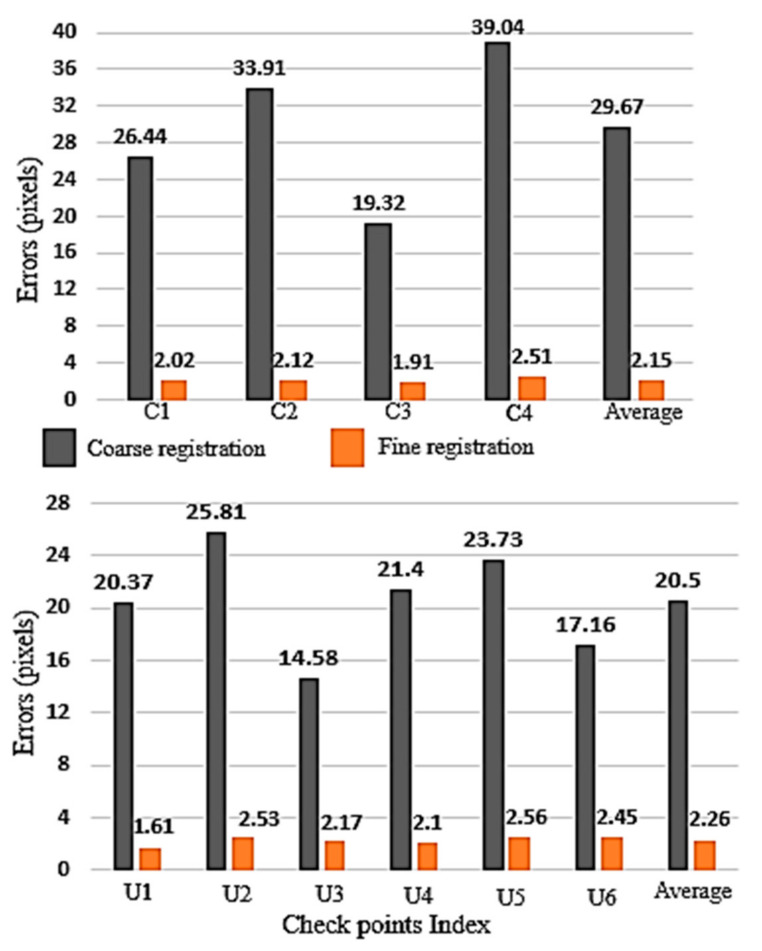
Total error values before and after applying coarse to fine registration method on close-range (**top**) and UAV (**bottom**) data.

In Figure 6, total errors of each checkpoint are compared before and after applying the fine registration method. The error values of the checkpoints for the close-range data have a variation between 1.19 and 2.51 pixels after fine registration, while these values have been about 19.32 to 39.04 pixels before fine registration. Also, total error values for the checkpoints on the UAV data have differences of about 1.61 to 2.53 pixels. The results show that these errors are in the range of 14.58 to 25.81 pixels before applying fine registration method.

As a result, maximum and minimum total error differences on the checkpoints are about within 1 pixel, which proves the stability of the photogrammetric triangulation and bundle adjustment based on the new proposed fine registration method.

The range error (i.e., Z) of the checkpoints is analyzed to completely assess the quality of the fine registration method. Table 3 shows the comparison between coarse and fine registration range error values. The minimum coarse registration range error value of the close range and UAV data are calculated to be about 18.5 and 10.6 pixels, which were altered to 1.7 and 1.01 pixels in the fine registration step, respectively. Maximum range error value on both datasets is estimated to be about 38 pixels, while it is improved to about 2 pixels after using the fine registration.

Likewise, average and RMSE range error value before applying the fine registration method for the close-range data are 28.95 and 29.89 pixels and for the UAV data are about 19.25 and 19.81 pixels, respectively. These values are improved to about 2 pixels after applying fine registration method.

**Figure 7:**
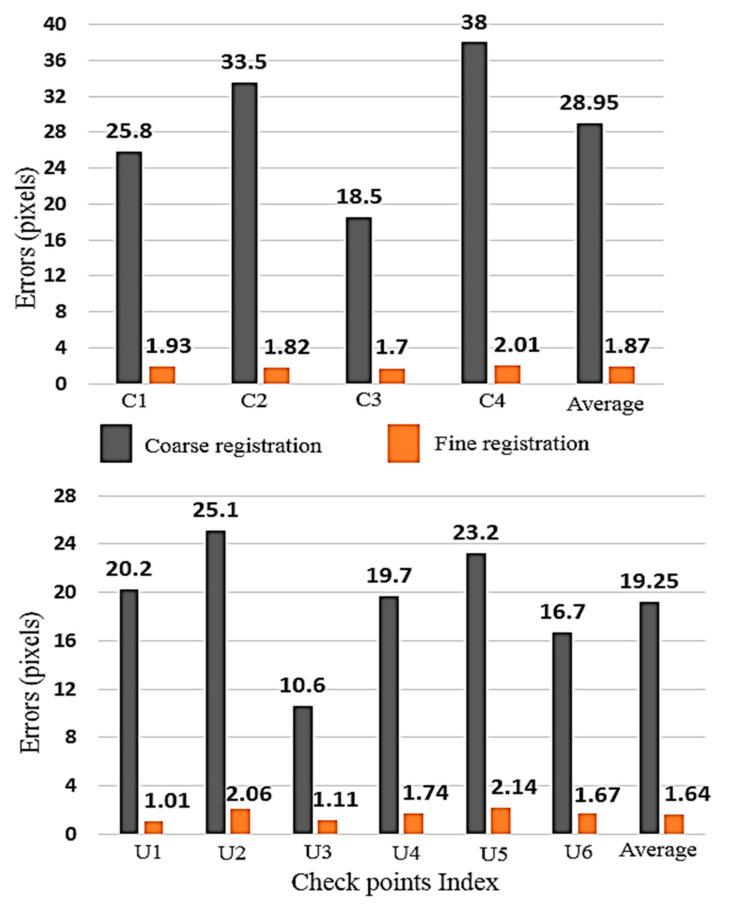
Range (Z) error values before and after applying coarse to fine registration method on close-range (**top**) and UAV (**bottom**) data.

Figure 7 illustrates the coarse and fine registration range error distribution on the checkpoints. Error ranges of all checkpoints after applying fine registration were reduced below 2.14 pixels. Meanwhile, the minimum range error for coarse registration on the checkpoints is 10.6 pixels for U3. There are approximately 20 pixels error variations on the checkpoints of the close-range and approximately 15 pixels for the UAV data in the coarse registration step, indicating broad deformation of the photogrammetric model. In addition, after using the fine registration, parameters such an error variation and model deformation are decreased below 1 pixel. As a result, our fine registration method not only improved the imagery and point cloud data alignment and calibration accuracy but also decreased the deformations of the photogrammetric model approximately underneath 1 pixel.

Additionally, checkpoints C2 in the close-range data and points U1 and U6 in the UAV data, which are far from the center of the photogrammetric model and images perspective centers, have range errors less than average range error values. Such a result proves the high quality of the fine registration method and the quality of the camera calibration and network design.

For further analysis, laser scanner cloud points were back-projected to the imagery data in two different situations. First, the coarse registration parameters (i.e., IOPs, EOPS, and Aps) are used for back projection. Second, fine registration parameters are employed to project back the point cloud points on the images.

**Figure 8:**
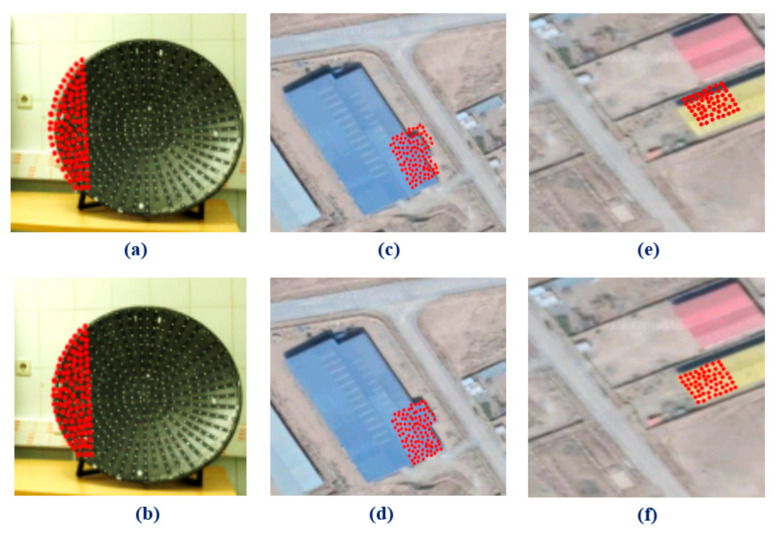
Back projection of the sub-area laser scanner points to the photogrammetric imagery (**a**,**c**,**e**) before fine registration; (**b**,**d**,**f**) after fine registration.

Because of the high density of the laser scanner points and for better representation of the results, point cloud data were resampled and fewer numbers of points were used. Also, we picked three different groups of the points on the objects that are easily distinguished from surrounding objects points. The first group of the points is related to a curved object that has height differences with background points. These points belong to the close-range data and can be distinguished easily on the imagery and point cloud data. Figure 8a shows misalignment of the point cloud and imagery data, while after applying fine registration method two datasets are correctly overlapped (Figure 8b).

Two other groups of the points pertain to UAV data and contain building rooftop points. Figure 8c,e represents misregistration of the point cloud and imagery data. After applying the new fine registration method, the alignment of the datasets is corrected (Figure 8d,f).

The results show the apparent biases between the imagery and back-projected laser scanner point cloud data before the fine registration iterative calculations. These biases are removed by the iterative fine registration calculations.

**Figure 9:**
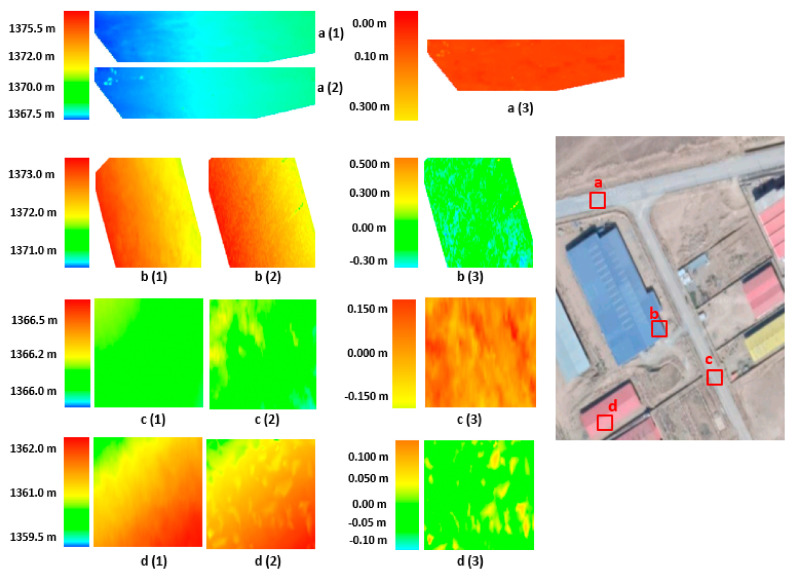
Comparison between photogrammetric DEM (**a** (**1**), **b** (**1**), **c** (**1**), **d** (**1**)) and laser scanner DEM (**a** (**2**), **b** (**2**), **c** (**2**), **d** (**2**)) and difference images (**a** (**3**), **b** (**3**), **c** (**3**), **d** (**3**)) for UAV data.

**Figure 10:**
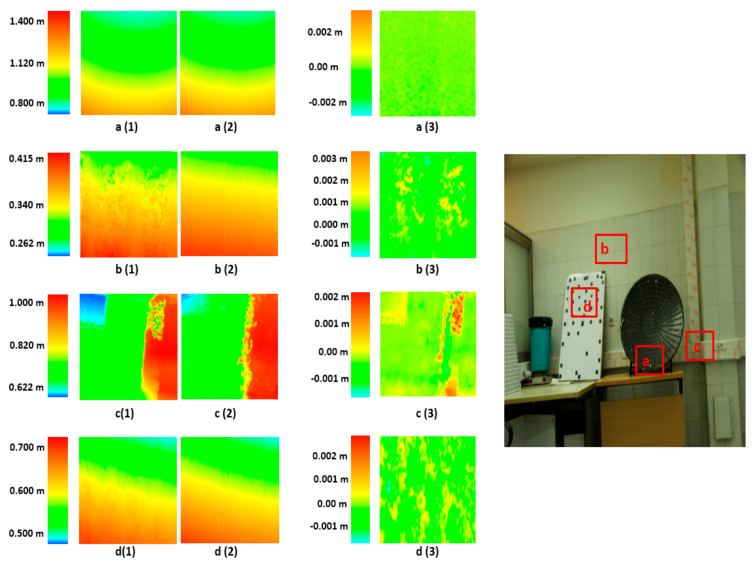
Comparison between photogrammetric DEM (**a** (**1**), **b** (**1**), **c** (**1**), **d** (**1**)) and laser scanner DEM (**a** (**2**), **b** (**2**), **c** (**2**), **d** (**2**)) and difference images (**a** (**3**), **b** (**3**), **c** (**3**), **d** (**3**)) for close range data.

To better examine the obtained results, we compare the photogrammetric point cloud and laser scanner point cloud. For this purpose, the Semi Global Matching (SGM) [40] method is used to generate a dense point cloud from photogrammetric images. The photogrammetric point cloud is estimated based on fine calibrated IOPs, Aps, and EOPs values. Then, the Digital Elevation Model (DEM) is generated for both photogrammetric point cloud and laser scanner point cloud. Afterward, to estimate difference image between the two-point clouds, the photogrammetric DEM and laser scanner DEM are subtracted and a new comparison grid map is estimated (Figure 9 and Figure 10).

Figure 9a (1) to Figure 9d (1) show the photogrammetric DEM of the four different parts of the UAV data for the study area. Also, Figure 9a (2) to Figure 9d (2) present the laser scanner DEM of the same parts. The resolution of the DEM data is 0.005 m. Further, Figure 9a (3) to Figure 9d (3) illustrate the difference image between photogrammetric and laser scanner DEM data. The area ‘a’ is an asphalt surface on the street and the difference image shows small values approximately under 1 pixel. Area ‘c’ is a dirt street and the difference values are about 3 pixels. Also, areas ‘b’ and ‘d’ are building surfaces, for which the difference values are about 2 pixels. By analyzing the images, it is seen that the difference between two surfaces is in the range of the fine registration accuracy, while in some points the differences are larger. These large values can occur due to dense matching errors or interpolation of the point cloud data for generating the DEM data.

Furthermore, Figure 10a (1) to Figure 10d (1) illustrate the photogrammetric DEM of the four different areas of the close-range data in the study area. Likewise, Figure 10a (2) to Figure 10d (2) show the laser scanner DEM of the same areas. The resolution of the close-range study area DEM data is 0.1 m. Additionally, Figure 10a (3) to Figure 10d (3) show the difference image produced by photogrammetric and laser scanner DEM data. Similar to the UAV results of the difference image, the difference between two DEM is in the range of the fine registration accuracy, while in some points, the differences are larger, and in others, they are smaller.

Comparison of the new methods with the comparable works is a routine procedure to show the effectiveness of the proposed new method. So, we compared our method with the proposed method of the authors of the [28]. In Table 4, total maximum error, average error, and RMSE values of our method are compared with one of the recently published articles having tested on both close range and UAV data.

The maximum error value changes from 2.12 to 2.51 pixels for those authors, while for our proposed method, it varies in the range of 2.51 to 2.57 pixels. Further, the average error value is between 2.15 and 2.26 pixels by our method, but between 1.89 and 2.01 pixels for those authors. The RMSE value of other authors is approximately in the range of 1.96 to 2.14 pixels while in our method is about 2.25 to 2.28 pixels (Table 4). Our maximum error, average error, and RMSE values are approximately equal to those of the recently published fine registration methods.

Moreover, our novel proposed method was examined on the different accuracies of the coarse registration values. These various coarse registration values for IOPs, EOPs, and APs were established by modifying them manually. As a result, we found that the proposed method works on coarse registration accuracies better than 35 pixels error (i.e., total error). The other analysis of this work is that such an analysis has not been reported elsewhere.

All the experiments are done on a desktop PC, with a 7-core processor paced at 4 GHz, 32 GB RAM.

## 5. Conclusions

This paper presents a novel method for coarse to fine registration of the photogrammetric imagery and laser scanner point cloud dataset. The method works based on minimizing the distances of the tie object space image points to the laser scanner point cloud data surface. The method not only employs the advantages of the photogrammetric model deformation exact correction without any ground control points, lines, and planes, but also demonstrates a wide range of applicability for coarse-to-fine registration of the various type of the point cloud and imagery data. Instead of conventional mathematical modelling, our method benefits from a physical interpretational modelling plan in which one shift constraint equation are applied on image and point cloud differential planes. This strategy converts our method into a hybrid feature based coarse-to-fine registration strategy via a combined bundle adjustment. The real experimental study tests on two different indoor and outdoor data proved the likely improved accuracy of registration. Therefore, the proposed fine registration method is an accurate and practical tool for registration of the different point cloud and imagery data. However, it must be noted that we do not claim that our method could work in the multipart green regions such as forest areas. In future works, it is required to alter our approach to a complete calibration and alignment method, which controls the local density of tie points based on surface curvature variations. Also, for simultaneous calibration, it proposes to correct multi-station terrestrial laser scanner point cloud systematic errors in the imagery and point cloud registration bundle adjustment.

## Figures and Tables

**Table 1:** Dataset specialization.

Sensors	Cameras			Laser Scanners	
**Sensor Type**	**Canon 7D**	**DJI Camera Sensor**	**Sensor Type**	**ScanStation2**	**RIEGL VMX-250**
**Site**	**Close-range**	**UAV**	**Site**	**Close-range**	**UAV**
**Distance to object (m)**	**4–6**	**80**	**Scan speed (points per second)**	50,000	600,000
**GSD (m)**	**0.001**	**0.025**	**Points density (points per square meter)**	180,000	40,000
**Number of images**	**14**	**37**	**Range accuracy (mm)**	5	5

**Table 2:** Comparison to coarse and fine registration total (XYZ) accuracy.

Error Type (Study Area)	Coarse Registration Accuracy (pixel)		
	Maximum Error	Average Error	RMSE
**Total (XYZ)** **(Close-range)**	39.04	29.67	30.60
**Total (XYZ)** **(UAV)**	25.81	20.5	20.85
**Error Type** **(Study Area)**	**Fine Registration Accuracy (pixel)**		
	**Maximum Error**	**Average Error**	**RMSE**
**Total (XYZ)** **(Close-range)**	2.51	2.15	2.25
**Total (XYZ)** **(UAV)**	2.57	2.26	2.28

**Table 3:** Comparison to coarse and fine registration range (Z) accuracy.

Error Type (Study Area)	Coarse Registration Accuracy (pixel)		
	Maximum Error	Average Error	RMSE
**Range (Z)** **(Close-range)**	38	28.95	29.89
**Range (Z)** **(UAV)**	25.1	19.25	19.81
**Error Type** **(Study Area)**	**Fine Registration Accuracy (pixel)**		
	**Maximum Error**	**Average Error**	**RMSE**
**Range (Z)** **(Close-range)**	2.01	1.87	1.88
**Range (Z)** **(UAV)**	2.14	1.63	1.68

**Table 4:** Comparison between our method and other authors for the fine registration accuracy (unit: Pixel).

Author Name	Study Site	Maximum Error	Average Error	RMSE
**Our Method**	**Close range**	2.51	2.15	2.25
	**UAV**	2.57	2.26	2.28
**Huang & et al.** [28]	**Close range**	2.52	2.01	2.14
	**UAV**	2.12	1.89	1.96

## Data Availability

We did not report any data.
